# The Late-Stage Protective Effect of Mito-TEMPO against Acetaminophen-Induced Hepatotoxicity in Mouse and Three-Dimensional Cell Culture Models

**DOI:** 10.3390/antiox9100965

**Published:** 2020-10-09

**Authors:** Mohammad Abdullah-Al-Shoeb, Kenta Sasaki, Saori Kikutani, Nanami Namba, Keiichi Ueno, Yuki Kondo, Hitoshi Maeda, Toru Maruyama, Tetsumi Irie, Yoichi Ishitsuka

**Affiliations:** 1 Department of Clinical Chemistry and Informatics, Graduate School of Pharmaceutical Sciences, Kumamoto University, 5-1 Oe-honmachi, Chuo-ku, Kumamoto 862-0973, Japan; shoebbmb@gmail.com (M.A.-A.-S.); dstcomrelat119@gmail.com (K.S.); 141p1014@st.kumamoto-u.ac.jp (S.K.); 140p1032@st.kumamoto-u.ac.jp (N.N.); 157p1004@st.kumamoto-u.ac.jp (K.U.); ykondo@kumamoto-u.ac.jp (Y.K.); tirie@gpo.kumamoto-u.ac.jp (T.I.); 2 Program for Leading Graduate Schools “HIGO (Health Life Science: Interdisciplinary and Glocal Oriented) Program”, Kumamoto University, 5-1 Oe-honmachi, Chuo-ku, Kumamoto 862-0973, Japan; 3 Department of Biochemistry and Molecular Biology, School of Life Sciences, Shahjalal University of Science and Technology, Sylhet 3114, Bangladesh; 4 Department of Biopharmaceutics, Graduate School of Pharmaceutical Sciences, Kumamoto University, 5-1 Oe-honmachi, Chuo-ku, Kumamoto 862-0973, Japan; maeda-h@kumamoto-u.ac.jp (H.M.); tomaru@gpo.kumamoto-u.ac.jp (T.M.)

**Keywords:** liver injury, acetaminophen, Mito-TEMPO, mitochondria, CCAAT/enhancer binding protein homologous protein, c-jun N-terminal kinases, endoplasmic reticulum, ER stress

## Abstract

An overdose of acetaminophen (APAP), the most common cause of acute liver injury, induces oxidative stress that subsequently causes mitochondrial impairment and hepatic necroptosis. N-acetyl-L-cysteine (NAC), the only recognized drug against APAP hepatotoxicity, is less effective the later it is administered. This study evaluated the protective effect of mitochondria-specific Mito-TEMPO (Mito-T) on APAP-induced acute liver injury in C57BL/6J male mice, and a three dimensional (3D)-cell culture model containing the human hepatoblastoma cell line HepG2. The administration of Mito-T (20 mg/kg, i.p.) 1 h after APAP (400 mg/kg, i.p.) injection markedly attenuated the APAP-induced elevated serum transaminase activity and hepatic necrosis. However, Mito-T treatment did not affect key factors in the development of APAP liver injury including the activation of c-jun N-terminal kinases (JNK), and expression of the transcription factor C/EBP homologous protein (CHOP) in the liver. However, Mito-T significantly reduced the APAP-induced increase in the hepatic oxidative stress marker, nitrotyrosine, and DNA fragmentation. Mito-T markedly attenuated cytotoxicity induced by APAP in the HepG2 3D-cell culture model. Moreover, liver regeneration after APAP hepatotoxicity was not affected by Mito-T, demonstrated by no changes in proliferating cell nuclear antigen formation. Therefore, Mito-T was hepatoprotective at the late-stage of APAP overdose in mice.

## 1. Introduction

Acetaminophen (N-acetyl-*p*-amino phenol; Paracetamol; APAP) is an over-the-counter drug, mostly used as an analgesic and antipyretic for different age groups worldwide with some after-effects at therapeutic doses [1,2]. However, an accidental or intentional overdose of APAP can cause acute hepatotoxicity that progresses to acute liver failure (ALF) [3]. APAP hepatotoxicity is responsible for about 46% of all ALF cases in the United States, and 40 to 70% in the United Kingdom and Europe [4,5,6,7]. Hepatic CYP2E1 (cytochrome P450) converts absorbed APAP into a very unstable toxic compound N-acetyl-*p*-benzoquinoneimine (NAPQI), a glutathione (GSH) conjugate [8,9]. Following an overdose of APAP, hepatic GSH becomes depleted and the NAPQI concentration increases. The excessively unstable toxic metabolite NAPQI can bind to sulfhydryl groups of cellular and mitochondrial proteins (for example, glutathione peroxidases, ATP synthase α-subunit) [10,11]. Mitochondrial protein-NAPQI adducts induced mitochondrial oxidative stress by impairing mitochondrial respiration [12] and cellular protein-NAPQI adducts activated JNK, which amplified mitochondrial protein-NAPQI-induced reactive oxygen species (ROS) generation [13,14]. Although the mechanism of mitochondrial oxidative stress in APAP-induced liver injury is poorly understood, mitochondrial oxidative stress is probably caused by electrons leaking from the electron transport chain (ETC) [15,16]. Moreover, the progression of hepatic injury involves multiple mediators including ROS, activated c-jun N-terminal kinase (JNK), C/EBP homologous protein (CHOP) induction, peroxynitrite, nitrotyrosine formation, and inflammatory mediators including interleukins (IL) and TNF-α [15,17,18,19,20,21,22].

Mitochondria are abundant in cellular systems and play a pivotal role by regulating energy metabolism, the cell cycle, ROS generation, and calcium homeostasis [23,24], and the mitochondrial activity and homeostasis of the redox state are crucial for controlling cellular survival and cell death pathways. Mitochondrial disorders generated by the metabolism of molecular oxygen and changes in the redox homeostatic nexus cause mitochondrial dysfunction [25]. Because mitochondrial dysfunction contributes to different common pathologies, mitochondria-targeted drug delivery is becoming an attractive mode of therapy [26]. Furthermore, mitochondrial oxidative stress-mediated mitochondrial dysfunction has a critical role in the development of APAP-induced acute liver injury and acute liver failure [27]. Therefore, drugs that have pleiotropic effects on mitochondria were identified as therapeutic agents against APAP-induced liver injury [28,29]. Moreover, mitochondria restoration or exogenous mitochondria might be an alternative treatment [30]. However, no mitochondria-specific antioxidant has been authorized for the attenuation of mitochondrial oxidative stress [25]. Mito-TEMPO (Mito-T) is a mitochondria-specific antioxidant [31] that has significant protective effects against APAP-induced acute liver injury [32,33].

In this study, we confirmed the protective effect of Mito-T against APAP-induced acute liver injury in mouse and human models. Mito-T contains a hydrophobic tetramethylpiperidine (TEMPO) group and lipophilic triphenyl phosphonium cation (TPP^+^) that are responsible for incorporation into mitochondria and scavenging mitochondrial superoxides, respectively [31]. The purpose of this study was to confirm the effectiveness of Mito-T against acute liver injury induced by an overdose of APAP, and the protective effect of Mito-T against APAP-induced mitochondrial oxidative stress, nitrotyrosine formation, and hepatic necroptosis in mice. We also observed the effect of Mito-T on liver regeneration after acute liver injury in mice. In addition, the effects of Mito-T against APAP-induced hepatocellular injury and mitochondrial oxidative stress were evaluated in a human HepG2 three-dimensional cell culture model.

## 2. Materials and Methods

### 2.1. Experimental Materials

Mito-TEMPO (Mito-T), acetaminophen (APAP), N-acetyl-L-cysteine (NAC), SP600125-JNK inhibitor, and Solution HS-15 were procured from Sigma-Aldrich Corporation (St. Louis, MO, USA). Concanavalin A (ConA), carbon tetrachloride (CCl_4_), corn oil, 10% formalin neutral buffer solution, and dehydrated dimethyl sulfoxide (DMSO) were procured from Wako Pure Chemical Industries, Ltd. (Osaka, Japan). Dulbecco’s modified Eagle’s medium containing a low glucose concentration (DMEM), penicillin/streptomycin (Pen Strep), and 2.5% trypsin (10×) was purchased from Gibco^®^ Life Technologies (Life Technologies Japan, Tokyo, Japan). Fetal bovine serum (FBS) was purchased from Biowest (Nuaillé, France). A cell counting kit (WST8) was obtained from Dojindo Laboratories (Kumamoto, Japan). MitoSOX™ Red mitochondrial superoxide indicator was purchased from Invitrogen (Thermo Fisher Scientific, Waltham, MA, USA). NanoCulture Plate MH pattern Low-binding 96 well Ver. 2 (Code No. NG-PLH9010) was purchased from MBL International Corporation^®^ (Woburn, MA, USA). All additional reagents and solvents utilized in this study were of reagent grade. Distilled and de-ionized bio-pure grade water was used in this study.

### 2.2. Animals and Protocols

We used the previously reported APAP-induced hepatic injury model in this study [34,35,36]. For in vivo experiments, 7–8-week-old male wild type C57BL/6J Jcl mice (CLEA Japan Inc., Tokyo, Japan) were used. The APAP and ConA-induced liver injury mouse models were prepared by dissolving APAP (400 mg/kg), and ConA (12.5 mg/kg) in phosphate buffered saline (PBS) to give a dose of 1 mL and 0.625 mL per 50 g body weight of C57BL/6J mouse, respectively. Dissolved APAP was heated to about 60 °C and cooled to 40–50 °C before intraperitoneal administration, whereas the ConA solution was administered intravenously. The CCl_4_ induced liver injury mouse model was prepared by dissolving CCl_4_ (0.025 mL/kg) in corn oil to a dose of 0.5 mL per 50 g of C57BL/6J mouse body weight and then administered intraperitoneally. All experimental animals were kept under controlled conditions (12-h light/dark cycle at 24 °C) with free access to food and water. We followed the guidelines of the Committee for Ethics on Animal Experiments of Kumamoto University (Approval numbers A27-131, A29-132, and A2019-102) for all our animal experiments and animal handling.

### 2.3. Drug Administration

Mito-T (2–20 mg/kg) was suspended in saline and injected intraperitoneally into mice at a dose of 0.5 mL per 50 g of mouse body weight. NAC (600 mg/kg) was suspended in saline to a dose of 0.6 mL per 50 g of mouse body weight and administered intraperitoneally.

### 2.4. Measurement of Alanine Aminotransaminase (ALT) Activity

Mice were anesthetized by the inhalation of isoflurane (Wako Pure Chemical Industries Ltd., Osaka, Japan) and then euthanized during sample collection. After laparotomy with a scalpel, a 1 mL syringe and 26 G × 1/2″ (0.45 × 13 mm) injection needle (Terumo^®^ Corporation, Tokyo, Japan) were used to collect blood from the abdominal vena cava. Whole blood samples were kept for 30–50 min at room temperature, and then centrifuged (3000× *g*, 10 min, 4 °C). Then, the supernatant of serum samples was collected and serum ALT levels were determined according to the supplier’s guidelines using a commercially available SPOTCHEM™ IIGPT/ALT assay kit (REF 77170) and automated clinical analyzer SPOTCHEM™ EZ SP-4430 (ARKRAY, Inc., Kyoto, Japan) [34].

### 2.5. Hepatic Histopathology

Liver histopathology was performed according to a previously described protocol [34]. In brief, histopathological specimens were prepared by fixing livers isolated from mice with 10% formalin neutral buffer solution followed by paraffin embedding using common methods. We cut 3-μm thick microtome sections and hematoxylin-eosin (H&E) staining was performed to observe necrotic cell death in APAP overdose livers. Terminal deoxynucleotidyl transferase-mediated dUTP nick end labeling (TUNEL) staining of liver samples was performed to observe hepatocyte death using an ApopTag^®^ Peroxidase In Situ Apoptosis Detection Kit (Merck Millipore, Billerica, MA, USA) according to the protocol supplied by the manufacturer. Images of stained sections were captured by a KEYENCE BIOREVO BZ-9000 microscope (Keyence Corporation, Osaka, Japan).

### 2.6. Immunostaining

For immunostaining, liver tissue samples were prepared, and immunohistochemistry was performed as previously reported [36]. Histopathological specimens were prepared by fixing livers isolated from mice with 4% paraformaldehyde and then embedding them in paraffin. We cut 3-μm thick microtome sections and immunostained them with three different antibodies (CHOP antibody, nitrotyrosine antibody, and PCNA antibody). Images of the stained liver samples were captured using the KEYENCE BIOREVO BZ-9000 microscope. We took 10 randomly-chosen images from each liver sample and counted the number of positive cells in each image (Supplemental Figure S1). The number of CHOP positive cells is shown as the mean of 10 fields.

### 2.7. Hepatic Total Glutathione Content

Liver specimens were prepared and the total glutathione (tGSH) level was estimated by the previously demonstrated technique [36,37]. Briefly, liver tissue samples were weighed after collection and stored (−80 °C) until tGSH was estimated. Then, tissue homogenates were suspended in 5 mL of 5% metaphosphoric acid solution at a ratio of 1:5 (*w/v*) and centrifuged for 15 min at 8000× *g* at 4 °C. The supernatant was collected and the tGSH concentration was determined by BIOXYTECH GSH/GSSG-412 (OXIS Health Products, Inc, Portland, OR, USA). Absorbance was measured using a spectrophotometer (V-530, JASCO Corporation, Tokyo, Japan) and the total GSH content was expressed as nmol/mg tissue.

### 2.8. Western Blotting

Protein expression was estimated by western blotting as previously reported [34,36]. In brief, 4 h after the administration of APAP, liver samples were excised, balanced to 150 mg, and kept in 0.5 mL of RIPA mix (490 μL of radio-immunoprecipitation assay (RIPA) buffer (Wako Pure Chemical Industries Ltd.), 5 μL of Halt™ protease inhibitor cocktail (Thermo Fisher Scientific) and 5 μL of Halt™ phosphatase inhibitor cocktail (Thermo Fisher Scientific)). Liver samples were homogenized using Micro Smash™ MS-100R (TOMY MEDICO., LTD, Tokyo, Japan) at 4000 rpm using zirconia and stainless-steel beads, and the supernatant was collected for quantitative BCA protein measurement as well as a protein extract solution. The protein samples were subjected to separation by SDS-polyacrylamide gel electrophoresis (SDS-PAGE) using 0.5 M/1.5 M Tris-HCl, 40% acrylamide, 10% sodium dodecyl sulfate, tetramethylethylenediamine (Sigma-Aldrich) and 0.1% Tris Buffered Saline (TBS-T). After separation, proteins from the gel were transferred onto a polyvinylidene difluoride (PVDF) membrane (Millipore Corp., Burlington, MA, USA) by Transblot (Bio-Rad Laboratories Inc., Berkeley, CA, USA). After blocking the membranes with 5% skim milk, they were washed three times with TBS in Tween-20, and incubated overnight in diluted primary antibody at 4 °C. Primary antibodies specific for CYP2E1 (Abcam Inc., Cambridge, MA, USA), *p*-JNK, JNK, and *β*-actin (Cell Signaling Technology, Danvers, MA, USA) were diluted at 1:1000. The PVDF membrane was rinsed three times with TBS in Tween-20 and stained with complementary secondary antibodies conjugated to anti-rabbit IgG (Cell Signaling Technology) (1:3000). To detect the antibody staining, SuperSignal^®^ West Pico Chemiluminescent Substrate (Thermo Scientific) was used, and the emission signal was visualized using Image Quant LAS-4000 (GE Healthcare Japan Corporation, Tokyo, Japan).

### 2.9. Hepatic mRNA Isolation and Real-Time RT-PCR

The liver specimens were balanced, chilled, and kept in RNA*later*™ solution (Invitrogen, Thermo Fisher Scientific) until assayed [35,37]. To isolate mRNA from the hepatic tissue samples, the liver tissues were homogenized using Micro Smash™ MS-100R (Tomy Medico Ltd.) in TRIzol^®^ reagent (Invitrogen™-Life Technologies, Tokyo, Japan), zirconia, and stainless-steel beads according to the guidelines provided by the supplier. The RNA concentration of each sample was determined using an Eppendorf BioSpectrometer^®^ kinetic (Eppendorf AG, Hamburg, Germany) and the samples were diluted to 200–2000 nM using RNase free water. cDNA libraries were prepared from the extracted hepatic RNA samples by using a High Capacity cDNA Reverse Transcription Kit (Applied Biosystems-Life Technologies, Tokyo, Japan). Quantitative real-time RT-PCR for mouse CHOP protein (*Chop*; NM_007837) and β-actin (NM_007393) was carried out using the following primers: CHOP forward, AGCTGGAAGCCTGGTATGAGGA, and reverse, AGCTAGGGACGCAGGGTCAA; β-actin forward, CATCCGTAAAGACCTCTATGCCAAC and reverse, ATGGAGCCACCGATCCACA. Real-time PCR analysis used diluted cDNA samples and Fast SYBR^®^ Green master mix (Applied Biosystems-Life Technologies Japan). The ΔΔCt method of StepOne™ Real-Time PCR System (Applied Biosystems-Life Technologies Japan) was used. A melting curve was generated to determine specificity. The correlative magnitude of the target gene mRNA was determined as fold induction normalized to the β-actin level (endogenous control).

### 2.10. Cell Cultures and Induction of the APAP Hepatotoxicity Model in 3-Dimensional HepG2 Cells

The cell culture and in vitro studies were performed according to our previous report [35]. Briefly, HepG2 (RIKEN BioResource Center, Tokyo, Japan), a human hepatoblastoma cell line, was cultivated at 37 °C, under 95% air and 5% CO_2_, in DMEM medium containing 10% FBS, 100 U/mL penicillin, and 100 U/mL streptomycin in a SANYO CO_2_ incubator (Marshall Scientific, Hampton, NH, USA). For sub-culture and seeding in NCPs, the confluent culture was rinsed with PBS (pH 7.4), disassembled with 0.25% trypsin, and centrifuged to collect the cells. For the 3D-cell culture, 96-well NCPs were used, and the cells were seeded at a density of 1 × 10^4^ cells/well in 100 µL. To prepare the 3D-cell culture model, 50 μL of DMEM medium was added to each well in 96-well NCPs and incubated for 10 min. Then, a cell suspension at twice the desired concentration was prepared and 50 µL of the cell suspension was added to the plate.

### 2.11. Cell Viability Assays in the 3D-HepG2 Cell Model

To evaluate APAP-induced cytotoxicity, we measured mitochondrial dehydrogenase activity in the 3D NCP-HepG2 cell culture using the WST-8 assay and Cell Counting Kit (Dojindo Laboratories) according to the supplier’s method. HepG2 cells were seeded at a concentration of 1 × 10^4^ cells/well in 96-well NCPs for 5 days. After spheroid formation, the cells were treated with 15 mM APAP, 15 mM APAP + 10 µM of Mito-T, and 15 mM of APAP + 100 µM of NAC, for 48 h at 37 °C in 5% CO_2_. The reagents were dissolved in DMEM and the control group was treated with medium only. Then the cells were incubated with 10 µL of WST-8 reagents for 180 min. WST-8 treated samples were transferred to a 96-well cell-culture plate (Greiner Bio-One) and samples were measured at an absorbance of 450 nm using a microplate reader (Tecan Co., Ltd., Männedorf, Switzerland). The protective effect of Mito-T and NAC were measured by combination treatment on APAP-treated HepG2 cells. The percentage of cell viability was estimated compared with the untreated controls.

### 2.12. Measurement of Mitochondrial Oxidative Stress in the 3D-HepG2 Cell Model

Mitochondrial oxidative stress was evaluated using a Mito-SOX™ Red mitochondrial superoxide indicator (Thermo Fisher Scientific). HepG2 cells were cultivated in 96-well NCPs at 3.0 × 10^4^ cells/well until spheroid formation and the spheroids were observed approximately within 72 h. Then, the 3D-HepG2 cells were treated by the following conditions: Control group (medium only), APAP group (15 mM of APAP), APAP + Mito-T group (15 mM of APAP + 10 µM of Mito-T), and APAP + NAC group (15 mM of APAP + 100 µM of NAC). The reagents were dissolved in DMEM and the control group was treated with medium only. After 24 h exposure to the reagents, the cells were treated with 5 µM of MitoSOX in DMEM for 15 min in the dark condition. Then, the medium containing MitoSOX was removed and replaced with PBS containing 5% FBS. The cells were immediately imaged at excitation/emission wavelengths of 490/510 nm and under bright field using a microscope (BIOREVO BZ-9000; Keyence Co., Osaka, Japan). Random fields of each well were analyzed by microscopy and the quantification of MitoSOX fluorescence was performed using ImageJ software (1.47v, National Institutes of Health, Bethesda, MD, USA). The data were expressed as fluorescence intensity per field of cell occupied area.

### 2.13. Statistical Analysis

All the experimental data are the mean ± standard error. Statistical analyses were performed using GraphPad Prism ver. 5.01 (GraphPad Software, San Diego, CA, USA). For comparisons between two groups, the F-test was used to test the population’s equal variance. The Student’s *t*-test was performed for equal variance and Welch’s *t*-test was used for unequal variance. Non-parametric multiple comparisons were performed if the variance was not uniform and Kruskal–Wallis analysis was performed after confirming significant differences (*p* < 0.05). Comparisons among three or more groups were made by the multiple comparison test. Statistical significance among different groups was analyzed by one-way analysis of variance (ANOVA) after the identification of uniform variance by Bartlett’s analysis (*p* < 0.05). When significant differences (*p* < 0.05) were observed, the data were analyzed further by Tukey’s (or Tukey–Kramer) multiple range test as appropriate. For the therapeutic time window experiment data, two-way ANOVA was performed. When significant differences (*p* < 0.05) were identified, data were analyzed further using the Bonferroni method.

## 3. Results

### 3.1. Protective Effect of Mito-T against APAP-Induced Liver Injury in C57BL/6J Mice

**Figure 1:**
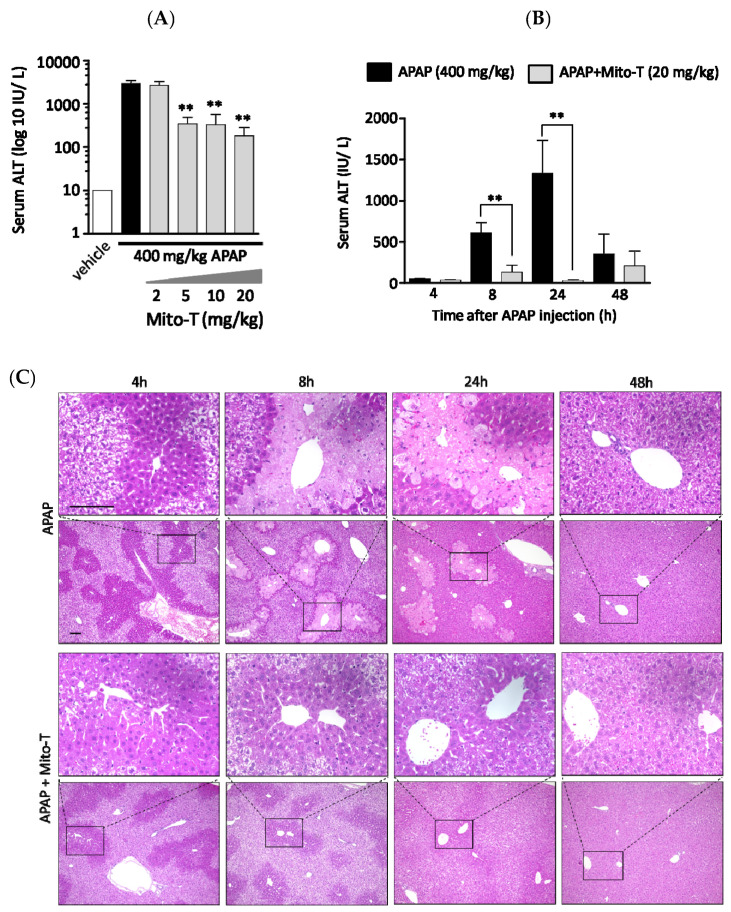
Mito-TEMPO (Mito-T) is hepatoprotective against acetaminophen (APAP) liver injury. (**A**) Mice were treated with 400 mg/kg APAP intraperitoneally, and then 2, 5, 10, or 20 mg/kg MT or saline was administered intraperitoneally 1 h later. The serum ALT activity was measured 24 h after APAP injection. Each bar represents the mean ± SEM of 3–5 mice. ** *p* < 0.01 vs. the APAP group. (**B**) Serum alanine aminotransaminase (ALT) activity was measured at 4, 8, 24, and 48 h after APAP (400 mg/kg, i.p.) injection. Mito-T (20 mg/kg, i.p.) and saline were administered 1 h after APAP administration. Each bar represents the mean ± SEM of 4–7 mice. ** *p* < 0.01 vs. the APAP group. (**C**) Representative hepatic sections from the APAP and APAP + Mito-T groups stained with H&E at 4, 8, 24, and 48 h after APAP administration.

A protective effect of Mito-T on liver injury was observed following an overdose of APAP in C57BL/6J Jcl wild type male mice. To confirm a dose dependent effect, four different doses of Mito-T (2, 5, 10, and 20 mg/kg) were administered intraperitoneally 1 h after a single APAP injection (400 mg/kg, i.p.). The hepatic injury biomarker transaminase level (ALT) in serum was measured 24 h after APAP treatment. We found that 5–20 mg/kg Mito-T significantly (*p* < 0.01) suppressed the APAP-induced increase in serum ALT levels whereas 2 mg/kg of Mito-T had no protective effect (Figure 1A). This indicated the effective dose of Mito-T was > 5 mg/kg, and 20 mg/kg of Mito-T was chosen for further experiments.

To observe the effect of Mito-T on APAP-treated mice, a time-course study of serum ALT activity was performed using eight groups containing 3–9 C57BL/6J mice per group. All eight groups were treated with 400 mg/kg APAP intraperitoneally, and then four groups were treated with Mito-T (20 mg/kg) and the other four groups were treated with saline (20 mg/kg) 1 h after APAP administration. The serum ALT activity was measured at 4, 8, 24 and 48 h after APAP injection. The ALT kinetic data showed that after APAP exposure, the ALT activity started to increase at 4 h and peaked at 24 h. Then, ALT activity declined to normal levels within 48 h. However, after Mito-T administration, the ALT level was increased slightly at 8 h and remained at a normal level at the other time points (Figure 1B). Representative H&E stained images showed the effect of APAP overdose (400 mg/kg, i.p.) on mouse liver was time dependent. The histological images of APAP-treated mouse livers indicated zonal necroptosis, also termed centrilobular necroptosis around the central hepatic vein after 24 h. Drug metabolizing enzymes were ubiquitously observed in the centrilobular area. In the Mito-T (20 mg/kg, i.p.) treated group, the H&E staining showed an alleviation of the effect of APAP (Figure 1C).

### 3.2. Efficacy of Mito-T on Liver Injury Progression in C57BL/6J Mice Following APAP Overdose

**Figure 2:**
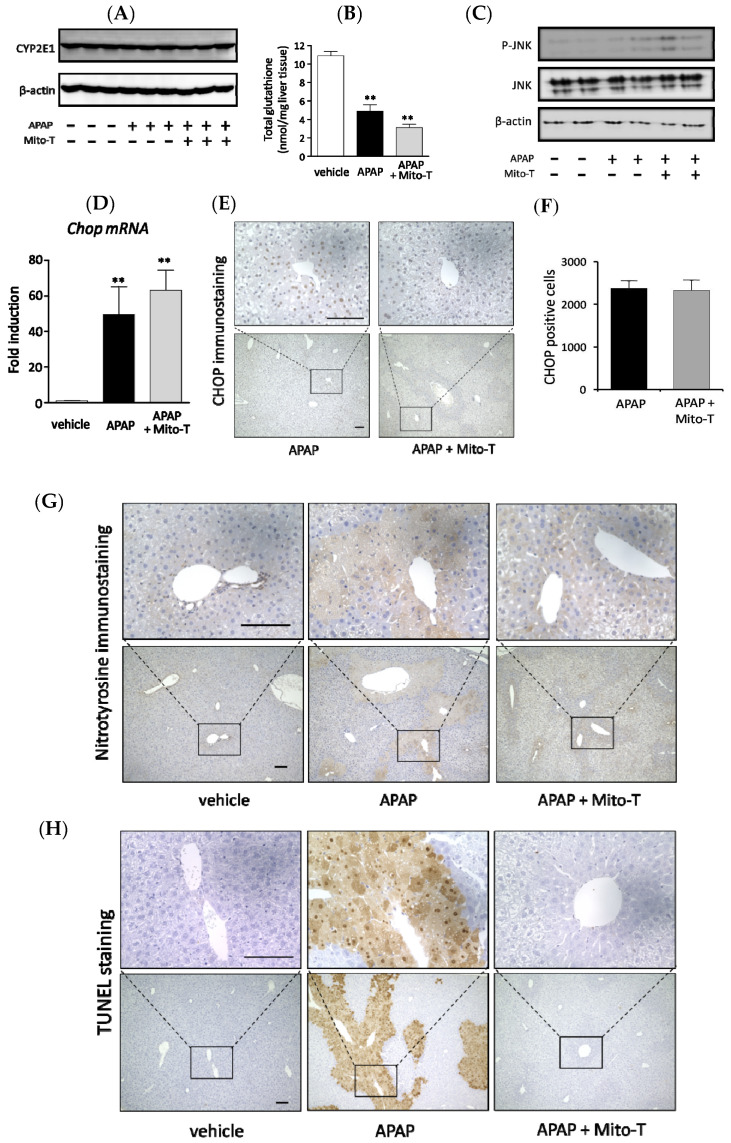
Effect of Mito-T on APAP liver injury pathophysiology. Mice were treated with APAP (400 mg/kg, i.p.) and 1 h later Mito-T (20 mg/kg, i.p.) or saline was administered. Liver tissue samples were collected from mice 4 h after APAP injection. Representative western blot of CYP2E1 expression (**A**) and total glutathione (GSH) level (**B**) in mouse liver is shown for each group. Each bar represents the mean ± SEM (*n* = 3–4). ** *p* < 0.01 vs. the saline group. (**C**) Representative western blot of *p*-c-jun N-terminal kinases (JNK) and JNK expression in mouse livers 4 h after APAP administration. (**D**) Liver tissue samples were collected from mice 4 h after APAP injection. mRNA levels of *Chop*in mouse livers were analyzed by quantitative real-time RT-PCR. (**E**) Representative CHOP staining of mouse livers is shown. (**F**) CHOP positive cells were quantified from CHOP immunostained mouse livers. Each value represents the mean ± SEM (*n* = 3–4). ** *p* < 0.01 vs. the saline group. (**G**) The liver samples were collected 8 h after APAP administration and nitrotyrosine immunohistochemical staining was performed. (**H**) The liver samples were collected 8 h after APAP administration and terminal deoxynucleotidyl transferase-mediated dUTP nick end labeling (TUNEL) immunohistochemical staining was performed.

APAP is metabolized to a cytotoxic compound NAPQI by the major drug-metabolizing enzyme CYP2E1. Then, NAPQI induces centrilobular hepatic damage and a reduction in the total glutathione (tGSH) level. In this study, we investigated whether there was any effect of Mito-T on the APAP metabolizing enzyme CYP2E1 and total GSH level in C57BL/6J mice. Mito-T (20 mg/kg, i.p.) was injected 1 h after APAP administration and samples were collected 4 h after APAP treatment. Whole liver western blotting showed that Mito-T did not affect CYP2E1 expression in the APAP-induced liver injury model (Figure 2A). Regarding the total glutathione level, a significant decrease was observed after the administration of APAP compared with the vehicle group; however, the decreased total glutathione amount was not alleviated by treatment with Mito-T (Figure 2B).

Activation of JNK in the hepatic cytosol is an early consequence of APAP-induced mitochondrial oxidative or nitrosative stress. Therefore, the effect of Mito-T on the expression of *p*-JNK was observed during APAP-induced liver injury progression. Mito-T (20 mg/kg, i.p.) was administered 1 h after APAP injection (400 mg/kg, i.p.) and liver tissue samples were collected 4 h after APAP administration. After protein extraction, *p*-JNK levels were measured by western blotting, which showed that APAP induced the expression of *p*-JNK; however, a suppressive effect was not observed in the Mito-T administered group (Figure 2C).

It was reported that the endoplasmic reticulum (ER) stress-inducible transcription factor CHOP induced cell death and was an important factor in the pathogenesis of different disease mouse models [19,38,39,40,41,42]. To investigate the effect of Mito-T on the expression of CHOP after APAP-induced liver injury, Mito-T (20 mg/kg, i.p.) was injected 1 h after APAP (400 mg/kg, i.p.) administration to C57BL/6J mice and tissue samples were collected 4 h after APAP injection. CHOP expression was measured by reverse transcription-polymerase chain reaction (RT-PCR) and immunostaining. The increased expression of *Chop* mRNA was observed after APAP treatment and a similar trend of increased *Chop* mRNA was found in the Mito-T group (Figure 2D). Furthermore, a similar increased expression pattern of CHOP positive cells was obtained for both groups (APAP and APAP + Mito-T) in CHOP immunostained mouse hepatic sections (Figure 2E,F). In addition, we observed that pretreatment with a JNK inhibitor, SP600125, significantly attenuated APAP-induced serum ALT levels confirming the involvement of JNK in the pathophysiology of our model (Figure S2).

Different types of mitochondrial ROS play a vital role in disease progression during APAP liver injury, where superoxide anions are the main reactive species converted to peroxynitrite by reacting with nitric oxide. Nitrotyrosine formation is a key mediator of peroxynitrite generation [43]. To confirm the effect of Mito-T on the APAP-induced hyper production of peroxynitrite in mitochondria, nitrotyrosine immunostaining was performed using an anti-nitrotyrosine antibody. An area stained with anti-nitrotyrosine antibody was observed in the APAP group but not in the APAP + Mito-T group (Figure 2G). Mitochondrial DNA fragmentation by DNA degrading enzymes during mitochondrial dysfunction was evaluated by the TUNEL staining assay. TUNEL positive cells were predominantly observed in liver sections of the APAP group, but very few were observed in the Mito-T treated group (Figure 2H). We also measured ROS generation in isolated mitochondria from mouse groups using MitoSOX and CM-H_2_DCFDA ROS detection probes. As shown in Supplemental Figure S3A,B, a significant increase in fluorescence intensity of the probes was observed in the APAP treated group compared with the vehicle group. Treatment with Mito-SOX significantly reduced this increase in fluorescence intensity.

### 3.3. Mito-T Prevents Cellular Injury and Reduces Mitochondrial Oxidative Stress in APAP-Treated HepG2 Cells

**Figure 3:**
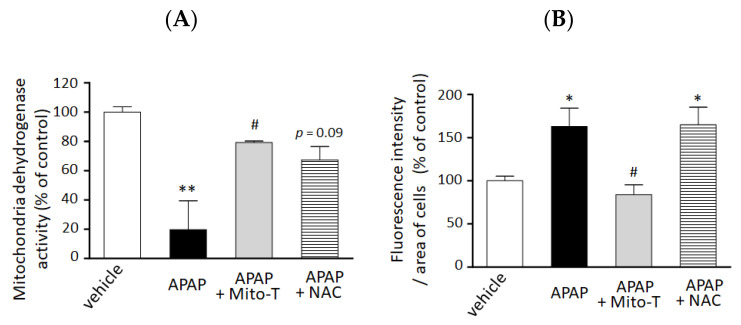
Preventive effect of Mito T and NAC against APAP-induced cellular injury and mitochondrial oxidative stress in a 3D cultured HepG2 cell model. HepG2 cells cultured in NCP (3D-NCP HepG2 cells) were exposed to 15 mM APAP in the presence or absence of Mito-T (10 µM) or NAC (100 µM). The reagents were added just after cell exposure to APAP. (**A**) Cell viability was measured 24 h after APAP exposure by changes in mitochondrial dehydrogenase activity using the WST-8 assay kit. Each bar represents the mean ± SEM (*n* = 3). (**B**) MitoSOX (5 µM) was added for 15 min after 24 h exposure to APAP. The cells were imaged at excitation/emission wavelengths of 490/510 nm and under bright field using a microscope. Random fields from each well were analyzed by microscopy, and the fluorescence of MitoSOX was quantified using ImageJ software (1.47v). Control or vehicle groups were treated with DMEM medium only. Each bar represents the mean ± SEM (*n* = 5). * *p* < 0.05, ** *p* < 0.01 vs. the vehicle group, # *p* < 0.05 vs. the APAP group.

We confirmed the hepatocellular protection against APAP hepatotoxicity by Mito-T in vitro. A significant decrease in cell viability was observed by 15 mM APAP exposure compared with vehicle treatment. Mito-T (10 µM) significantly alleviated the APAP-treated cytotoxicity in the 3D-NCP HepG2 cell culture (Figure 3A). The preventive effect of Mito-T was similar or greater than that of NAC (100 µM).

We also examined the efficacy of Mito-T on mitochondrial oxidative stress reduction in the 3D NCP HepG2 cell culture system. As shown in Figure 3B, treatment with Mito-T (10 µM) significantly reduced APAP (15 mM)-induced mitochondrial oxidative stress by MitoSOX whereas NAC (100 µM) co-treatment did not attenuate the increased oxidative stress induced by APAP.

### 3.4. Therapeutic Time Window of Mito-T and Its Role in APAP-Induced Liver Injury

**Figure 4:**
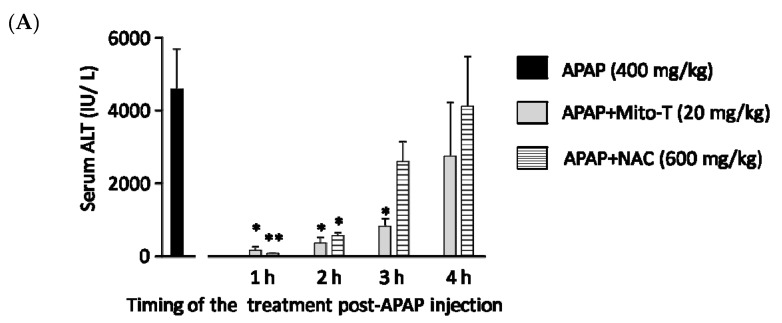

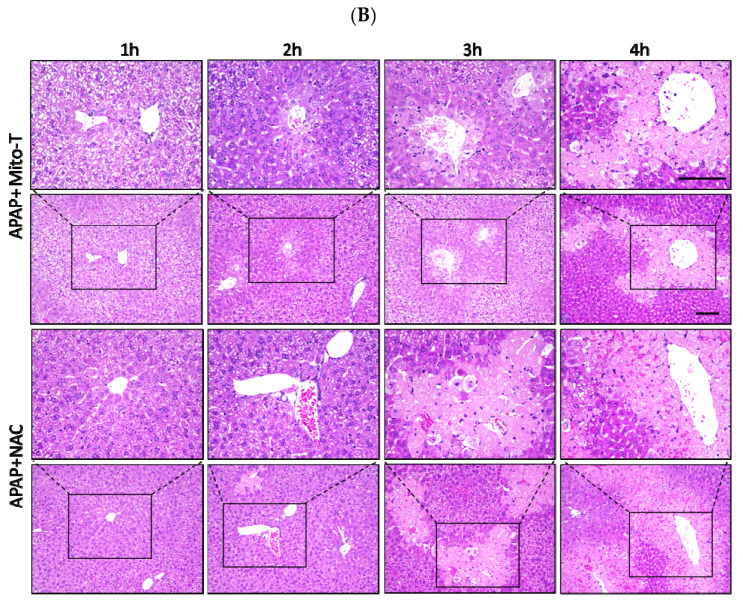
Therapeutic time window of Mito-T and NAC. Mito-T (20 mg/kg, i.p.) or NAC (600 mg/kg, i.p.) were administered at 1, 2, 3, and 4 h after APAP administration. Blood and liver tissue samples were collected 24 h after APAP (400 mg/kg, i.p.) injection. (**A**) Serum ALT activity was measured 24 h after APAP administration. (**B**) Representative hepatic sections from APAP+Mito-T and APAP+NAC groups were stained with H&E. Scale bar: 100 µm. Each value represents the mean ± SEM of 5–8 mice. * *p* < 0.05, ** *p* < 0.01 vs. the APAP group.

N-acetyl L-cysteine (NAC) is the only clinically approved therapeutic for APAP-induced liver injury. It was reported that NAC acts upstream of the pathogenesis process. Previously it was shown that NAC is effective for early presenting patients; however, most cases of APAP-induced liver injury are hospitalized after several hours [44]. This study confirmed that Mito-T effectively decreased the transaminase level after APAP hepatotoxicity and that it functioned downstream of injury progression compared with NAC. We investigated the therapeutic time window of Mito-T and NAC in the APAP-induced liver injury model and found that Mito-T treatment was effective and might therefore be a candidate treatment. It was observed that at 1, 2, and 3 h after APAP (400 mg/kg, i.p.) administration, Mito-T (20 mg/kg, i.p.) treatment significantly reduced the serum ALT level measured 24 h after APAP injection, whereas NAC (600 mg/kg, i.p.) administration only had a significant hepatoprotective effect at 1 and 2 h after APAP injection (Figure 4A). Representative H&E staining (Figure 4B) showed Mito-T and NAC had similar effects on APAP-induced liver necrosis.

### 3.5. Effect of Mito-T on Liver Regeneration after APAP-Induced Liver Injury

**Figure 5:**
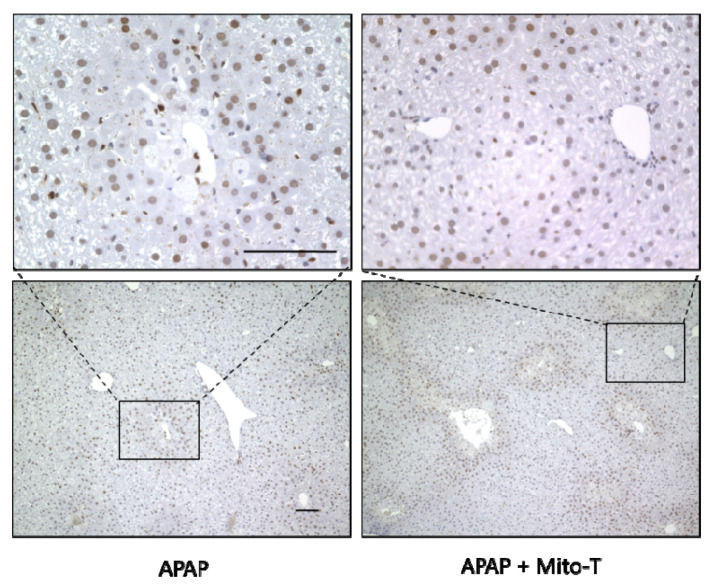
Effect of Mito-T on proliferating cell nuclear antigen (PCNA) expression. Mice were treated with Mito-T (20 mg/kg, i.p.) or saline 12 h after APAP (400 mg/kg, i.p.) administration. Liver tissue samples were collected 24 h after APAP injection. Representative PCNA staining of the hepatic sections in mice. Scale bar: 100 µm.

After APAP-induced liver injury, damaged hepatocytes are removed by autophagy and homeostasis is maintained by the division and proliferation of healthy cells [45]. Proliferating cell nuclear antigen (PCNA) is a protein identified in cells at the late G1 phase to the early DNA synthesis phase (S phase) after the division phase (M phase) and is used as a cell cycle marker [46]. In this study, Mito-T (20 mg/kg, i.p.) was administered 12 h after APAP (400 mg/kg, i.p.) administration, the liver was excised 24 h later, and PCNA immunostaining was performed. The APAP and APAP+Mito-T groups had similar levels of PCNA positive cells (Figure 5).

### 3.6. Effect of Mito-T on Other Drug-Induced Liver Injury Models

**Figure 6:**
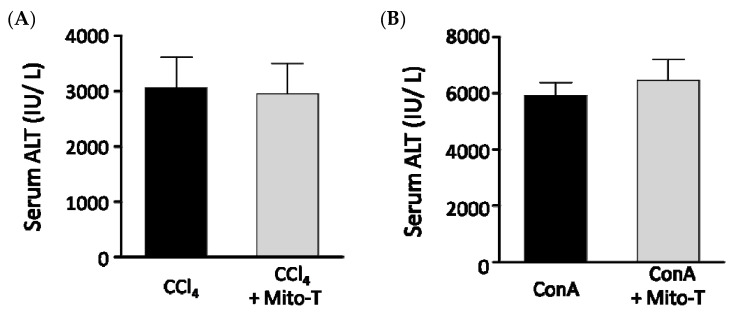
Effect of Mito-T on serum ALT levels in mice administered CCl_4_ or ConA. Mice were treated with Mito-T (20 mg/kg, i.p.) 1 h after CCl_4_ (0.025 mg/kg, i.p.) or ConA (12.5 mg/kg, i.v.) administration. The serum ALT activity was measured 24 h after CCl_4_ (**A**) or ConA (**B**) injection. Each value represents the mean ± SEM (*n* = 6–8).

Because Mito-T had hepatoprotective effects against APAP-induced liver injury, we investigated whether Mito-T had a protective effect against other fulminate hepatic injury models similar to APAP liver injury. Carbon tetrachloride (CCl_4_) is metabolized by CYP2E1 as is APAP and induces liver fibrosis by producing radicals [47]. In our study, Mito-T (20 mg/kg, i.p.) was administered 1 h after CCl_4_ (0.025 mL/kg, i.p.) administration and serum ALT values were measured 24 h after CCl_4_ administration. An increased serum ALT level was observed in the CCl_4_-treated group, which was not attenuated by Mito-T treatment (Figure 6A). Concanavalin A (ConA) also induces hepatotoxicity by activating immune cells such as CD4+ T cells and inducing the production of TNF-α, IL-6, IL-1β, and IFN-γ [48,49]. In this hepatopathy model, inflammatory responses induce hepatic injury related to the activation of JNK, which is also induced during APAP-induced hepatopathy. One hour after ConA (12.5 mg/kg, i.v.) administration, Mito-T (20 mg/kg, i.p.) was administered and serum ALT levels were measured 24 h after ConA injection. Serum ALT levels were not suppressed by Mito-T (Figure 6B).

## 4. Discussion

In this study, we confirmed the marked attenuation of APAP-induced serum ALT elevation and centrilobular necrosis in mice by Mito-T (5–20 mg/kg), which was dose- and time-dependent. We observed no effect on JNK and CHOP activation, CYP2E1 expression, and total GSH level. These data are similar to data reported by Du et al. [32,33]. We also demonstrated the attenuating potential of Mito-T in a human in vitro APAP 3D-HepG2 model [35]. In addition, we identified changes in PCNA expressions, and the therapeutic time-window in the mouse model. These data suggest that Mito-T is a potential therapeutic candidate to target mitochondrial oxidative stress.

JNK is a member of the mitogen-activated protein kinase (MAPK) family, which is activated by different stimuli, including drugs, ER stress, and ROS [50]. Following APAP administration, *p*-JNK translocates into mitochondria to induce opening of the mitochondrial permeability transition pore, which amplifies oxidative stress by mitochondrial ROS [15]. Although Mito-T has a significant protective effect, it does not attenuate p-JNK protein expression. CHOP plays an important role in APAP liver injury progression [19], and we observed that Mito-T significantly attenuated APAP-induced liver injury and liver necroptosis without inhibiting the APAP-induced increase in *Chop*mRNA expression and liver CHOP immunostaining in the mouse model. Furthermore, we observed that SP600125 pretreatment significantly attenuated serum ALT elevation and *Chop* mRNA expression following APAP administration. Based on these findings, we suggest that the activation of JNK and CHOP are upstream of the APAP hepatotoxicity pathophysiology and that Mito-T might act downstream of this mechanistic pathway (Figure S4).

A human model for the study of APAP hepatotoxicity is urgently required because of the limitations of in vivo studies using murine models. In this study, we used an NCP-3D culture of HepG2 cells as an in vitro human model, which we previously reported shows the key mechanistic features of APAP hepatotoxicity and might be used to evaluate the mechanism and prospective drug candidates for APAP-induced hepatic injury [35]. Data from the current study indicate that Mito-T has cytoprotective potential for APAP-induced cell death in the 3D-HepG2 cell culture model (Figure 3) and that Mito-T significantly alleviated APAP-induced mitochondrial oxidative stress in the NCP 3D HepG2 cell culture system (Figure 4). Furthermore, it can be suggested that the attenuation of mitochondrial oxidative stress by Mito-T is independent of the contribution from inflammatory cells such as neutrophils and Kupffer cells.

NAC is the only current antidote for APAP-induced liver injury, and it is effective in early presenting APAP hepatotoxicity patients [51]. However, most APAP hepatotoxicity patients present 24 h or later [52]. In this study, the late-stage protective effect of Mito-T against APAP-induced liver injury was observed at 3 h after APAP treatment in the mouse model. A wide therapeutic time window of Mito-T might overcome the limitations of NAC treatment following APAP overdose-induced liver injury. This confirms the speculation of the superior efficacy of Mito-T over NAC [32,33]. It was reported that although early treatment with novel therapeutics alleviated APAP-induced liver injury, their late administration deteriorated liver injury and impaired liver regeneration [53,54]. Therapeutic approaches for acute liver injury following an overdose of APAP should consider the liver regeneration mechanistic pathway. Damaged hepatocytes undergo autophagy for clearance or proliferation for repair, which require oxidative stress. Therefore, the oxidative stress induced by APAP liver injury in the late phase plays a vital role in liver regeneration. PCNA was observed in cells at the late G1 phase to early DNA synthesis phase (S phase) after the division phase (M phase) [46]. In the current study, we demonstrated that late treatment (12 h after APAP administration) with Mito-T did not inhibit PCNA expression following APAP-induced liver injury.

APAP overdose induces liver injury and hepatic cell death occurs by oncotic necrosis, necroptosis [55,56] or apoptosis [57,58]. A recent report suggested that following necrosis, limited secondary apoptosis was induced by Mito-T after APAP overdose in a mouse model [33]. Liver histopathology indicated that Mito-T significantly attenuated APAP-induced hepatic DNA damage, although hepatic swelling and vacuolization were still present. The current study suggests that Mito-T attenuates APAP-induced liver injury and DNA fragmentation significantly, although some limitations remain regarding its protective effect. Therefore, further study is required to investigate the mechanism of APAP-induced hepatocyte death.

In this study, we demonstrated the attenuating effects of Mito-T against APAP hepatotoxicity in mouse and cellular models. However, this study had some limitations. First, the precise mechanism of the hepatoprotective action of Mito-T against APAP hepatotoxicity was not fully clarified. In this study, Mito-T prevented liver damage related to elevated serum ALT levels, nitrotyrosine formation, and DNA fragmentation, without any changes in p-JNK and CHOP expression. However, we did not evaluate other MAPK and ER stress-related mediators, such as extracellular signal-regulated kinase [59], p38 MAPK [60], X-box binding protein 1 [61], or inositol-requiring enzyme 1α [61], which were reported to be important mediators in APAP hepatotoxicity. Further study is needed to investigate MAPK and ER stress-related mediators in the preventive mechanisms of Mito-T against APAP hepatotoxicity. Second, whether Mito-T can improve mitochondrial activity in hepatocytes during APAP hepatotoxicity is still unknown. Some reports demonstrated that APAP overdose reduced the activity of the electron transport chain during hepatotoxicity [27,62,63]. Although mitochondrial oxidative stress was evaluated using ROS detection probes and nitrotyrosine staining in mouse and cellular models in this study, we did not measure parameters that indicate mitochondrial activity, such as MitoTracker™, CMXRos fluorescence, isocitrate dehydrogenase-1 and 2, peroxisome proliferator-activated receptor γ coactivator-1α expression, or lactate dehydrogenase activity. We also examined mitochondria dehydrogenase activity using a WST-8 assay in the HepG2 cell system and found that Mito-T might attenuate the dysfunction of mitochondrial activity in APAP-exposed hepatocytes. However, further experiments are required to confirm this. Third, the effect of Mito-T on liver recovery from APAP intoxication, such as hepatocyte regeneration, and comparison of the effects with NAC were not fully identified. As mentioned above, we evaluated the effect of late-phase treatment with Mito-T during APAP liver injury using PCNA staining. However, other cell proliferation biomarkers, such as bromodeoxyuridine, cyclin D1, and Ki67, were not measured. In addition, we did not examine whether the normal tissue repair process occurred without abnormal healing such as remodeling, after Mito-T treatment. Indeed, Du et al. [33] suggested that Mito-T might limit secondary apoptosis, which may be important in the recovery process during late-phase liver injury. They also reported that an approved antidote NAC did not inhibit secondary apoptosis during the late phase of APAP hepatotoxicity. However, Yang et al. [64] reported that prolonged treatment with NAC significantly impaired liver regeneration during hepatotoxicity. Therefore, the effect of NAC on liver regeneration is controversial and further study to elucidate and compare the action of Mito-T and NAC in the repair process is warranted. Fourth, there is a critical difference in the cellular APAP liver injury model using 3D-cultured HepG2 cells and clinical APAP hepatotoxicity. For example, the concentration of APAP (15 mM) that induces significant cellular damage in 3D HepG2 cells was approximately >5 times higher than the blood levels of APAP in patients with APAP intoxication [65]. This difference is a common limitation in the cellular models of APAP hepatotoxicity, including primary cultured hepatocytes [54,66] and HepaRG cells [67,68]. Despite these limitations, we previously demonstrated that the 3D HepG2 cell model was similar to APAP liver injury with regards to NAPQI production, GSH depletion, and JNK pathway activation, indicating it might be a useful model to screen drug candidates for APAP hepatotoxicity [35]. Furthermore, the results from cellular experiments suggested that Mito-T protected hepatocytes independent from the influence of immune cells such as Kupffer cells and neutrophils. Finally, the time course of APAP hepatotoxicity progression was distinct from that of clinical patients with APAP liver injury. Indeed, the progression of live injury after APAP overdose tends to be much faster in mice than in humans. Previous studies reported that the maximum serum ALT elevation and centrilobular necrosis were observed between 12 and 24 h in mice [69] and 36–48 h in humans after an overdose of APAP [44]. When considering the therapeutic time-window of Mito-T and NAC, we cannot ignore the “species-dependent effect” in mice. Although the mouse model is considered the most representative model of liver injury in APAP intoxication patients, further study is warranted to demonstrate the therapeutic time-window of Mito-T and NAC in other models, using human hepatocytes, such as primary culture cells or HepaRG cells. Nevertheless, this study reports the beneficial action of Mito-T in APAP hepatotoxicity models and the critical role of mitochondrial oxidative stress in the development of APAP hepatotoxicity.

## 5. Conclusions

The protective effect of Mito-T against APAP-induce liver injury was confirmed in this study using mouse and human models. Mito-T markedly attenuated the APAP increase in transaminase levels, mitochondrial oxidative stress, nitrotyrosine formation, hepatic DNA damage, and hepatic necroptosis. This study confirmed the specific protective mechanism of Mito-T following APAP hepatotoxicity and its effectiveness in liver regeneration. We suggest that Mito-T is a potential therapeutic candidate that targets mitochondrial oxidative stress and which can function in the late phase of APAP-liver injury when compared with NAC, although additional studies are necessary to confirm this.

## Supplementary Materials

The following are available online at https://www.mdpi.com/2076-3921/9/10/965/s1, Figure S1. Quantification of CHOP positive cells in immunohistological images. Figure S2. CHOP induction in APAP liver injury and the effect of a JNK inhibitor. Figure S3. Mito-T reduces APAP-induced mitochondrial oxidative stress in mouse livers. Figure S4. Pathophysiology of APAP-induced liver injury.
