# Supplementary material for: The Late-Stage Protective Effect of Mito-TEMPO against Acetaminophen-Induced Hepatotoxicity in Mouse and Three-Dimensional Cell Culture Models
Source: Antioxidants (Basel). 2020 Oct 9;9(10):965. doi: 10.3390/antiox9100965 (PMC7601533; doi:10.3390/antiox9100965)
Supplement: Supplementary file 1 [file antioxidants-09-00965-s001.pdf]

Supplementary Data

FS1. Quantification of CHOP positive cells in immunohistological images.

To investigate the effect of Mito-T on APAP-induced increased CHOP protein expression in the mouse model, saline or Mito-T (20 mg/kg, *i.p.*) was administered 1 h after APAP (400 mg/kg, *i.p.*) injection and tissue samples were collected 4 h after APAP administration. Anti-CHOP antibody was used for CHOP immunostaining. CHOP positive cells were quantified in ten random fields of each immunostained sample as shown in Supplemental Figure 1S. A similar increased expression pattern of CHOP was observed for both groups (APAP + saline and APAP + Mito-T) in CHOP immunostained hepatic sections. Quantitative data showed no significant difference in CHOP expression between APAP + saline and APAP + Mito-T groups.

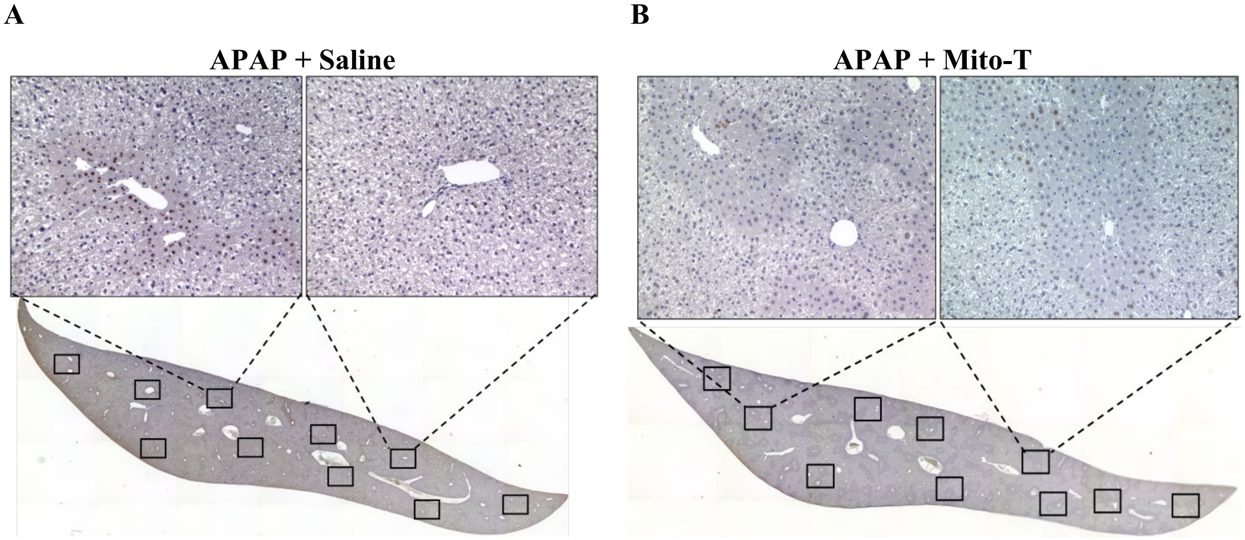

Figure S1. Effect of Mito-T on APAP-induced CHOP induction. Mice were treated with APAP (400 mg/kg, *i.p.*) and 1 h later Mito-T (20 mg/kg, *i.p.*) or saline was administered. Liver tissue samples were collected from mice 4 h after APAP injection. We took 10 random images from each liver sample and quantified the CHOP positive cells.

FS2. Chop induction in APAP liver injury and the effect of JNK inhibitor.

JNK activation is the early consequence of APAP-induced mitochondrial oxidative or nitrosative stress. To confirm the involvement of JNK, SP600125 a JNK inhibitor (30 mg/kg, *i.p.*) was administered 1h before APAP (400 mg/kg, *i.p.*) injection. After 8h of the APAP administration, the blood and tissue samples were collected. Serum ALT level was estimated and *Chop* mRNA level was analyzed by quantitative RT-PCR. SP600125 significantly attenuated the APAP liver injury (Fig. S2A) and *Chop* mRNA expression in C57BL/6J mice. (Fig. S2B).

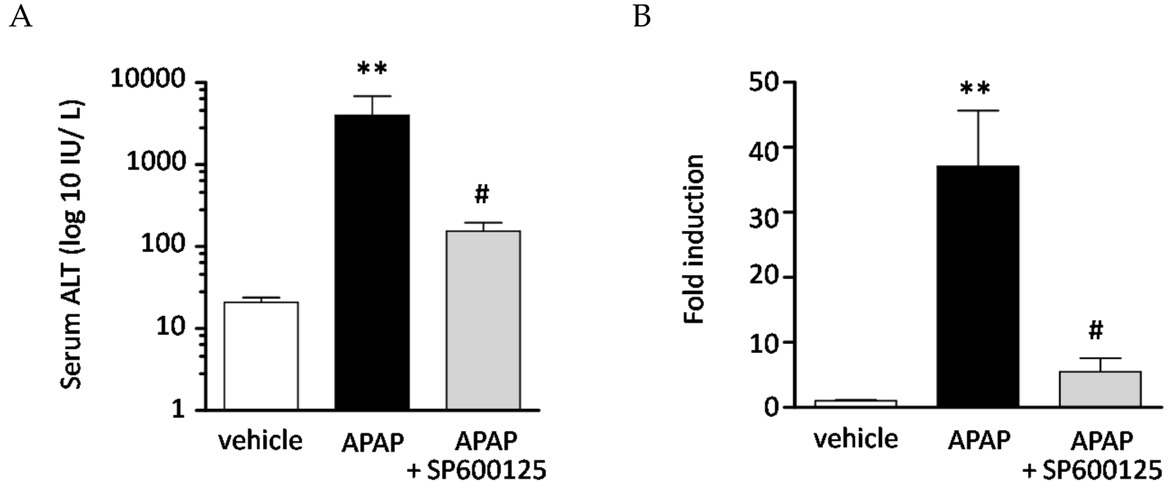

Figure S2. Effects of a JNK inhibitor SP600125 on APAP-induced serum ALT elevation (A), and hepatic Chop mRNA expression in mice (B). The mice were treated with APAP (400 mg/kg, *i.p.*) and 1h before SP600125 (30 mg/kg, *i.p.*) or saline was administered. SP600125 was dissolved in HS-15 solution, and HS-15 solution used as a vehicle. Blood samples were collected 8h after APAP injection from mouse and the serum ALT activity measured. Each value represents the mean ± SEM. (n = 5). $^{**}p<0.01$ *vs.* vehicle group and $\#p<0.05$ *vs.* APAP group.

FS3. Mito-T reduces APAP-induced mitochondrial oxidative stress in mouse livers.

Following the overdose of APAP, excessive unstable reactive metabolite NAPQI was generated and bound to mitochondrial and cellular proteins. In the mitochondria, oxidative and nitrosative stresses were generated by APAP-induced activated JNK. Therefore, the effect of Mito-T (20 mg/kg, *i.p.*) on mitochondrial oxidative stress was estimated using a mitochondrial-specific ROS detection fluorescent probe, Mito-SOX (Figure S3A) and a CM-H_2DCFDA probe (Figure S3B) following APAP (400 mg/kg, *i.p.*) injection. A single dose of Mito-T markedly attenuated the APAP-induced increased ROS in mouse livers and its efficacy on mitochondrial ROS scavenging was confirmed.

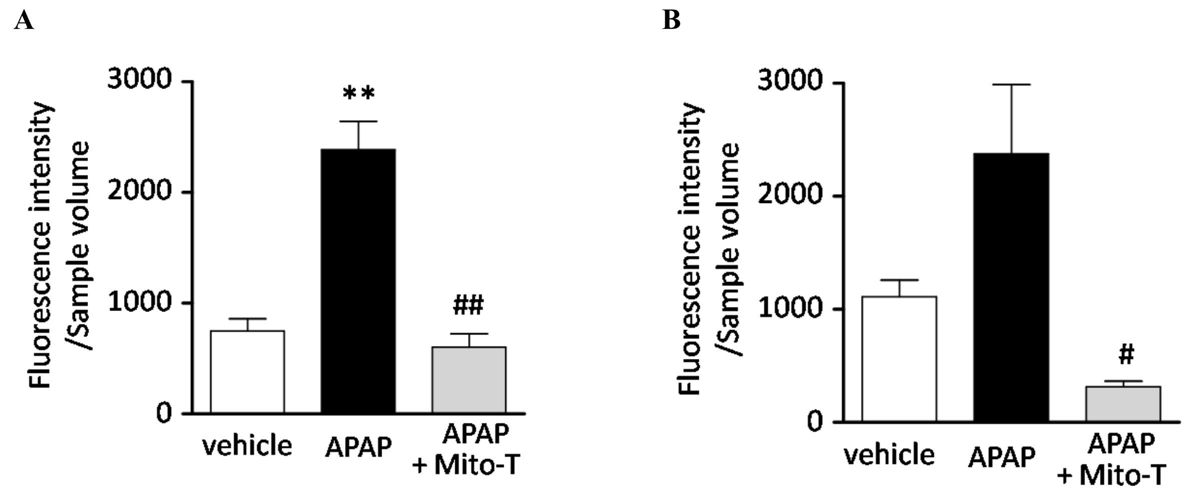

Figure S3. Effect of Mito-T on mitochondrial ROS generation in mouse livers. Mouse liver samples were collected 4 h after APAP (400 mg/kg, *i.p.*) injection. Mito-T (20 mg/kg, *i.p.*) was added 1 h after APAP administration. Mitochondria were isolated from mouse livers and mitochondrial ROS generation was estimated using Mito-SOX and CM-H_2DCFDA probes. The fluorescence was measured 10 min after Mito-SOX (A) and CM-H_2DCFDA (B) addition. Each value represents the mean ± SEM. (n = 3–4). $^{**}p < 0.01$ vs vehicle group, and $^{\#}p < 0.05$ vs APAP group.

FS4. Pathophysiology of APAP-induced liver injury.

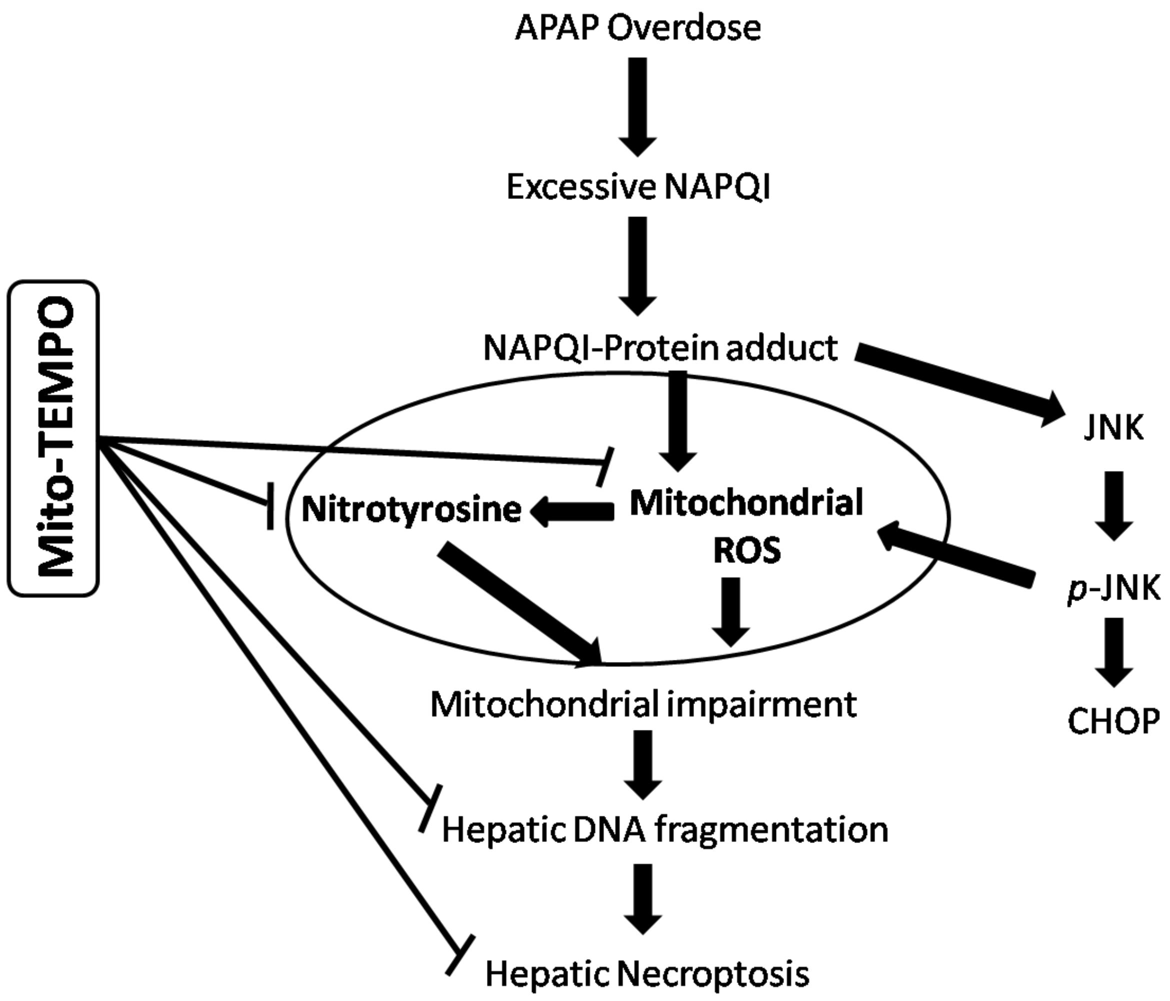

Figure S4. Schematic representation of APAP pathophysiology and action point of Mito-T.

Supplementary (Materials and Methods)

S 1. *Mitochondrial ROS determination*

Mitochondrial oxidative stress was measured using MitoSOX and CM-H2DCFDA. For this study, mitochondria were isolated from mouse livers using a commercially available Mitochondria Isolation Kit for Tissue (Thermo Fisher Scientific, Waltham, MA, USA) according to the supplier's protocol. For MitoSOX, isolated mitochondria were suspended in suspension buffer (125 mM KCl, 2 mM K_2HPO_4, 20 mM HEPES, 5 mM malic acid, 5 mM pyruvic acid, 4 mM $MgCl_2$, 3 mM ATP, 50 μM EGTA) and protein was quantitated by the BCA method. The amount of mitochondrial protein was adjusted to 200 μg, and then MitoSOX was added and incubated at 37°C for 5 min. The fluorescence (485 nm for excitation and 590 nm for emission) was measured by a microplate reader (Tecan Co., Ltd., Männedorf, Switzerland). For H_2-DCFDA, isolated mitochondria were suspended in suspension buffer (125 mM sucrose, 150 mM KCl, 10 mM HEPES-KOH, 2.5 μM rotenone, 5 mM KH_2PO_4, 5 mM succinate). The amount of mitochondrial protein was adjusted to 250 μg by the BCA method. Then, CM-H_2DCFDA was added and incubated at 37°C for 5 min. The fluorescence (485 nm for excitation and 530 nm for emission) was measured by a microplate reader.
